# Cost-effectiveness of atazanavir-ritonavir versus lopinavir-ritonavir in HIV patients initiating first-line antiretroviral therapy

**DOI:** 10.1186/1758-2652-13-S4-P234

**Published:** 2010-11-08

**Authors:** MS Broder, T Juday, J Uy, EY Chang, TGK Bentley

**Affiliations:** 1 Partnership for Health Analytic Research, Beverly Hills, USA; 2 Bristol-Myers Squibb, USP Medical, Plainsboro, USA

## Purpose of the study

Selection of initial antiretroviral therapy (ART) may be informed by factors such as efficacy, adverse effects, and cost. This study assessed the lifetime cost-effectiveness of atazanavir-ritonavir (ATV/r) versus lopinavir-ritonavir (LPV/r) in HIV patients initiating first-line ART.

## Methods

A Markov microsimulation model was developed to project lifetime health-related outcomes, costs, quality-adjusted life years (QALYs), and cost-effectiveness of ATV/r versus LPV/r, both with tenofovir-emtricitabine, as first-line ART. Virologic suppression, baseline characteristics, state transition probabilities, cholesterol changes, and adverse effects were based on 96-week CASTLE results. HIV-related mortality, opportunistic infection (OI) and AIDS rates, coronary heart disease (CHD) risk, treatment adherence, costs, and utilities were obtained from published sources. Costs were reported in 2009 US dollars. Sensitivity analyses were conducted to assess the robustness of study results.

## Summary of results

Compared with patients initiating LPV/r, patients initiating ATV/r were estimated to have longer time in first-line therapy, fewer cases of AIDS, OI, CHD, and diarrhea, more cases of hyperbilirubinemia (HB), and higher costs. While absolute survival was similar, patients initiating ATV/r were predicted to have longer quality-adjusted survival. Overall, ATV/r added 0.26 QALYs at a cost of $6,826, producing an ICER of $26,421 per QALY gained. Sensitivity analyses indicated that at a willingness to pay threshold of $50,000 per QALY, ATV/r was cost effective 94% of the time. Table 1

**Table 1:** 

Health-Related Outcomes							
	Time on First-Line Treatment (months)	AIDS cases (per 1000 patient years)	OI cases (per 1000 patient years)	CHD cases (per 1000 patient years)	Diarrhea cases (per 1000 patient years)	HB cases (per 1000 patient years)	Absolute Survival (life years)

LPV/r	70.7	20.054	0.519	5.511	6.262	0.247	18.51

ATV/r	97.3	19.081	0.443	5.437	1.272	6.986	18.52

Cost, QALY, and Cost-Effectiveness							

	Cost	Incremental Cost	Quality-Adjusted Survival (QALY)	Incremental QALY	Incremental Cost-Effectiveness		

LPV/r	$269,160	--	10.761	--	--		

ATV/r	$275,986	$6,826	11.020	0.258	$26,421		

## Conclusions

Accounting for both lifetime costs and QALYs, ATV/r is cost effective (less than $50,000 per QALY) compared with LPV/r in HIV patients initiating first-line ART.

